# Genetic characterisation of influenza B viruses detected in Singapore, 2004 to 2009

**DOI:** 10.1186/1756-0500-7-863

**Published:** 2014-12-01

**Authors:** Muhammad Raihan Jumat, Richard J Sugrue, Boon-Huan Tan

**Affiliations:** Division of Molecular and Cell Biology, School of Biological Sciences, Nanyang Technological University, Singapore, 637551 Republic of Singapore; Detection and Diagnostics Laboratory, Defence Medical and Environmental Institute, DSO National Laboratories, 27 Medical Drive, Singapore, 117510 Republic of Singapore; Saw Swee Hock School of Public Health, Faculty of Medicine, National University Singapore, Singapore, 117549 Republic of Singapore

**Keywords:** Influenza B, Phylogenetic analysis, Viral evolution, Surveillance, Singapore, Circulating lineage, Vaccine efficiency

## Abstract

**Background:**

Influenza B viruses are classified into two main lineages: Yamagata-like and Victoria-like, which differ antigenically and phylogenetically. To understand the evolution of influenza B viruses in South East Asia as well as to determine the vaccine efficacy, we genetically characterised gene segments 4, 6 and 8 from non-tissue culture adapted influenza B viruses detected in Singapore from 2004 to 2009.

**Methods:**

vRNA were extracted from the nasopharyngeal swabs or nasal washes of SAF servicemen displaying febrile and respiratory symptoms, and subjected to PCR assay to test for the presence of influenza B virus. The PCR-positive specimens were next subjected to sequencing of the full gene segments 4 (HA), 6 (NA/NB) and 8 (NS1/NEP). The nucleotide sequences were aligned together with that of other specimens isolated from South East Asia as well as the vaccine strains. Phylogenetic trees of each gene segment were constructed and the amino acid alignments were analysed.

**Results:**

A majority of the Singaporean specimens analysed in this study, from 2004–2009, had gene segment 4 from the Victoria-like lineage and gene segment 6 from Yamagata-like lineage. Some of these specimens had both gene segments from the Yamagata lineage and this resulted in several vaccine mismatches. Gene segment 8 from majority of these specimens clustered separately from both the Yamagata and Victoria strains. The HA protein of most of the Singaporean specimens isolated post 2000 contained a glycosylation site at position 211, which was not dominant prior to 2000. No amino acid substitution conferring drug-resistance was found in either the HA or NA proteins.

**Conclusions:**

The presence of both lineages co-circulating post 2000, suggests that a trivalent vaccine is not enough to confer immunity to the general public, strongly endorsing the inclusion of both lineages in the vaccine. Several amino acid substitutions were observed, prompting in depth functional analyses.

**Electronic supplementary material:**

The online version of this article (doi:10.1186/1756-0500-7-863) contains supplementary material, which is available to authorized users.

## Background

Influenza B viruses belong to the *Orthomyxoviridae* family and has a genome of 8 negative, single-stranded segments. Although Influenza B viruses circulates primarily in humans several reports had suggested that seals can serve as a possible animal reservoir for the virus [1–3]. The virus was initially isolated in 1940 [4] and since the early 1980s two distinct lineages have predominated; B/Victoria/2/87-like and B/Yamagata/16/88-like viruses [5, 6]. In the 1980s, the Victoria lineage was dominant and this was followed by the Yamagata lineage in the 1990s. Since the year 2000, both lineages have been detected at similar frequencies globally [7, 8].

The clinical symptoms associated with influenza B virus infection are generally similar to that of influenza A virus [9–11]. However, a few studies have shown that influenza B infections are linked with severe symptoms [10, 12–16]. Throughout 2012, influenza A virus infections dominated the total influenza cases reported globally and regionally by the Global Influenza Surveillance Response System (GISRS http://www.who.int/influenza/gisrs_laboratory/en/). Amongst influenza B infections, the GISRS reported that the Victoria-lineage caused more infections that the Yamagata-lineage in 2012 [17]. When analysed separately, the South East Asia region showed that influenza B virus was responsible for more than a third of all influenza infections. At the global level, influenza B viruses accounted for more than half of influenza infections between weeks 4–18 and 45–52 of 2012 [18]. Majority of these influenza B virus specimens isolated belonged to the Victoria lineage [19].

The two most abundant glycoproteins of influenza B viruses are hemagglutinin (HA) and neuraminidase (NA) [20]. Through the activity of both its subunits, the HA protein functions in receptor binding and in membrane fusion, facilitating viral entry [20, 21]. Unlike the HA protein, the NA functions late in the viral replication, cleaving the α–(2,3) and α–(2,6) glycosidic links between the terminal sialic acids moieties of glycoproteins, allowing for successful viral shedding [22, 23]. Both proteins present as structural epitopes which are recognised by the host immune system [24]. This selective pressure results in these proteins undergoing significant antigenic drift, resulting in antigenic variation from one epidemic to be different from the next. Genetic reassortment of the gene segments occur frequently between the two main influenza B lineages. This means that circulating viruses may have different combinations of gene segments [25, 26].

Singapore, being a tropical country, experiences a higher prevalence of influenza viruses than most temperate countries. Its tropical conditions as well as being a commercial hub has allowed for influenza viruses to circulate all year round with peaks between April-July and November-January [27–29]. Between the years of 1972–1999, there have been 25 reported influenza epidemics and four of which were caused by influenza B viruses. However, two out of these epidemics had an influenza A strain co-circulating with the predominant influenza B strain [29]. An epidemiological survey of respiratory infections amongst Singaporean military recruits found that up to a third of influenza infections were caused by influenza B infections [30]. The emergence of the pandemic H1N1 of swine origin in 2009 resulted in a suppression of influenza B virus circulation in Singapore. Since 2010, the prevalence of influenza B infections seems almost reciprocal to the prevalence of the influenza A viruses in Singapore [31]. For the past 5 years, a minimum of 20% of all influenza infections in Singapore were attributed to influenza B viruses [32]. It has been estimated that influenza B infections are responsible for 14.8 out of 100000 deaths in tropical countries yearly [28]. With the current trend in increasing resistance to treatment [33], and with an estimated of 50% vaccine efficacy in Singapore [34], this number is anticipated to increase.

This study aims to determine the nucleotide sequences of the circulating influenza B virus strains in Singapore between 2004 and 2009 which were not egg/tissue-culture adapted. By comparing the lineage identity of these strains to that of the vaccine strains, we would be able to observe for any mismatches which may result in inadequate immunological protection. Differences in the nucleotide and amino acid sequence would allow us to observe the evolutionary mechanisms of influenza B viruses and understand the divergence of the circulating strains from the vaccine strains. Furthermore, phylogenetic analysis of current and previously sequenced Singaporean strains can allow us to track the reassortment of influenza B virus over the last 50 years. Lastly, analysis of the mutation pattern of these circulating strains may provide greater insights in drug resistance and antibody recognition. We believe that this is the first study to genetically characterise non egg/tissue-culture adapted influenza B virus strains in Singapore. While the samples collected in this study were from the military, it is important to note that a large percentage of Singaporean males are enrolled into the military at the age of 18. This makes the results obtained in this study representative of national surveillance [30].

## Results

### Sequence and phylogenetic analysis of gene segments 4 and 6

**Figure 1:**
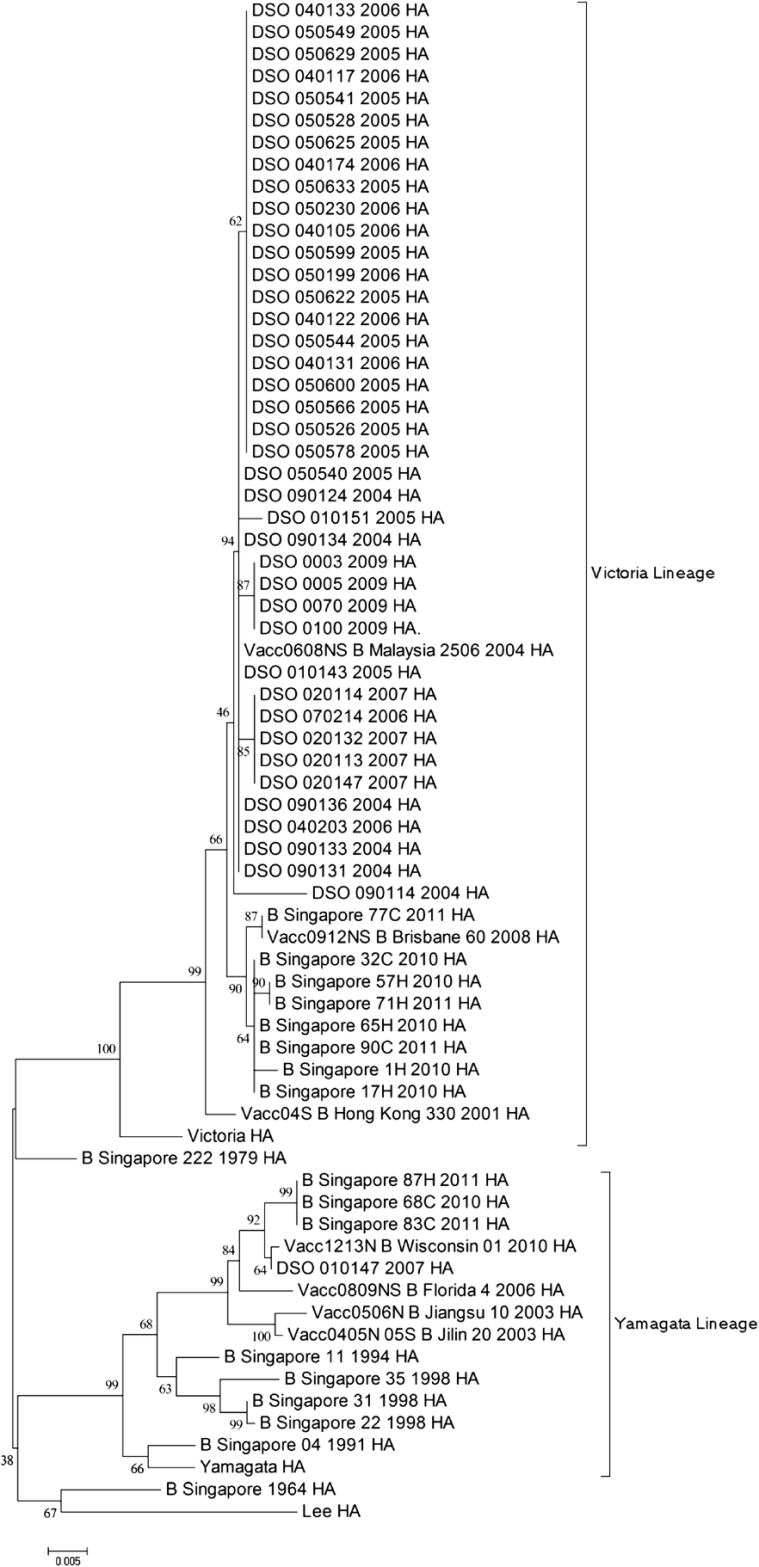
**Phylogenetic tree of the HA gene segment of Singaporean specimens isolated from 2004 to 2009.** The HA sequences of these specimens were aligned together with the HA gene segments of B/Lee/40, B/Yamagata/16/88, B/Victoria/2/87, the vaccine strains from 2004–2013 (Table 1) and the HA gene segments of Singaporean strains isolated in 1964, 1979, 1991, 1994, 1998, 2010 and 2011. Representative strains of influenza B from countries within SEA, such as Hong Kong, Philippines, Taiwan, Thailand, Malaysia and Myanmar, isolated post 2000 available from GenBank were included in this alignment as well. Lineages were marked by the labelled brackets.

**Figure 2:**
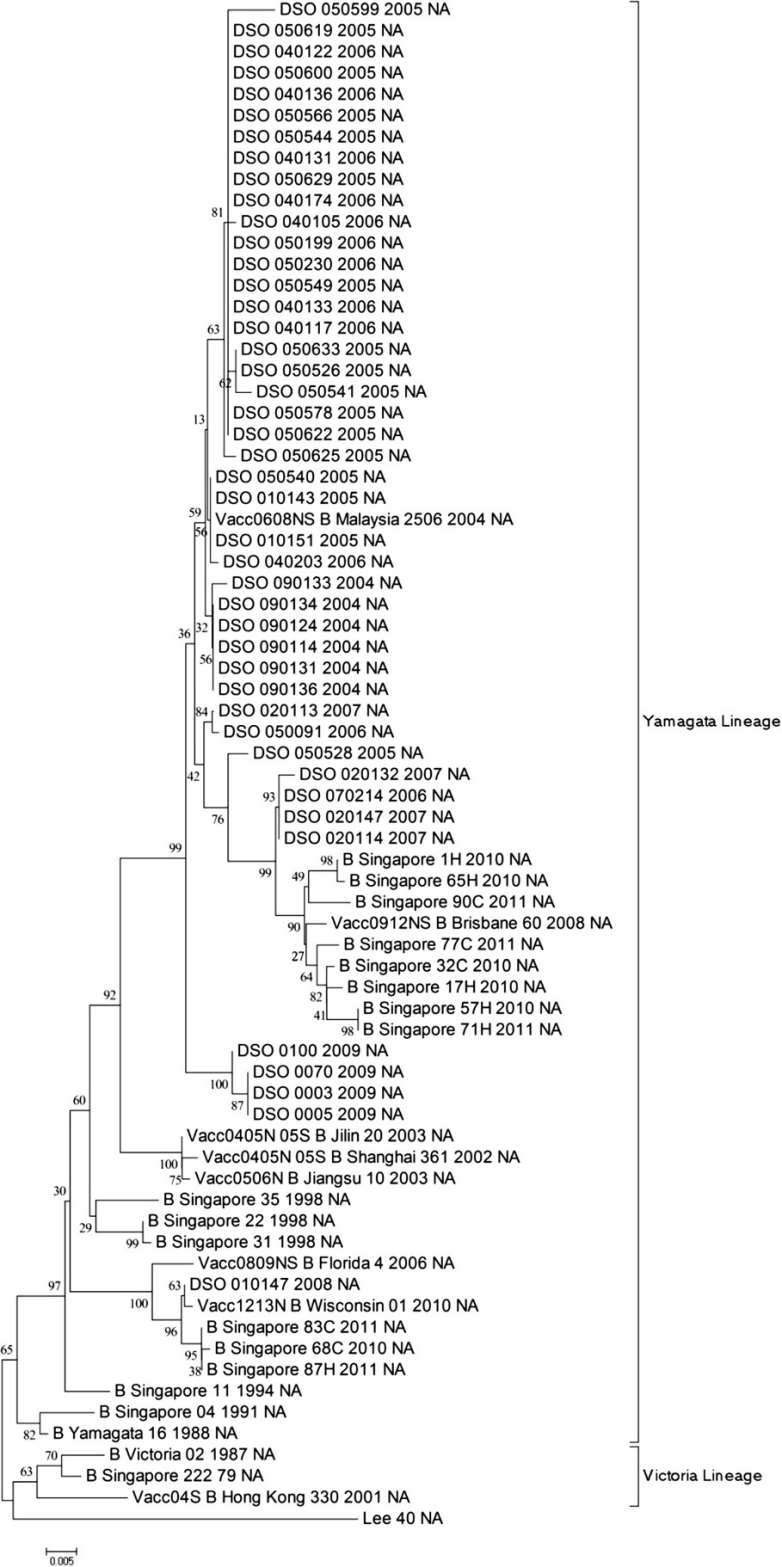
**Phylogenetic tree of the NA gene segment of Singaporean specimens isolated from 2004 to 2009.** The NA sequences of these specimens were aligned together with the NA gene segments of B/Lee/40, B/Yamagata/16/88, B/Victoria/2/87, the vaccine strains from 2004–2013 (Table 1) and the NA gene segments of Singaporean strains isolated in 1979, 1991, 1994, 1998, 2010 and 2011. Representative strains of influenza B from countries within SEA, such as Hong Kong, Philippines, Taiwan, Thailand, Malaysia and Myanmar, isolated post 2000 available from GenBank were included in this alignment as well. Lineages were marked by the labelled brackets.

Viral RNA (vRNA) was extracted directly from clinical specimens and used as template for PCR amplification and sequencing. A list of all the specimens sequenced in this study can be found in Additional file 1: Table S1. Three overlapping DNA fragments corresponding to the open reading frames of the HA and NA genes were PCR-amplified and sequenced. The resulting contig was then aligned with the rest of the clinical specimens as well as representative strains from SEA and the vaccine strains of 2004–2013 (Table 1) to produce the phylogenetic trees (Figures 1 and 2). Out of the 46 clinical specimens isolated in this study, the HA gene was sequenced from 42 while the NA gene from 44. The HA gene of the clinical specimens sequenced in this study displayed a similarity of 83.5%-99.9% while that for the NA gene; 84.5%-99.8%, when compared amongst themselves.

As shown in Figure 1, the gene segment 4 of the specimens sequenced in this study clustered within the Victoria-like lineage together with other recent Singapore strains, except for DSO_010147_2007. Generally, the specimens clustered according to their year of isolation. The specimen DSO_010147_2007 was isolated in year 2007, but clustered within the Yamagata lineage, together with some of the recent Singapore strains isolated in 2010 and 2011. Figure 2 shows the phylogenetic tree of gene segment 6 of the same specimens and shows that they fall within the Yamagata lineage. Similar to that in Figure 1, most of these specimens clustered according to their year of isolation (Figures 1 and 2).

**Table 1:** **Influenza B component of the bi-annual trivalent influenza vaccine (TIV) from 2004-2013**

Year	Hemisphere	Strain	Lineage	Abbreviation
2012-2013	Northern	B/Wisconsin/1/2010	Yamagata	Vacc1213N B Wisconsin 01 2010
2012	Southern	B/Brisbane/60/2008	Victoria	Vacc0912NS B Brisbane 60 2008
2011-2012	Northern	B/Brisbane/60/2008	Victoria	
2011	Southern	B/Brisbane/60/2008	Victoria	
2010-2011	Northern	B/Brisbane/60/2008	Victoria	
2010	Southern	B/Brisbane/60/2008	Victoria	
2009-2010	Northern	B/Brisbane/60/2008	Victoria	
2009	Southern	B/Florida/4/2006	Yamagata	Vacc0809NS B Florida 4 2006
2008-2009	Northern	B/Florida/4/2006	Yamagata	
2008	Southern	B/Florida/4/2006	Yamagata	
2007-2008	Northern	B/Malaysia/2506/2004	Victoria	Vacc0608NS B Malaysia 2506 2004
2007	Southern	B/Malaysia/2506/2004	Victoria	
2006-2007	Northern	B/Malaysia/2506/2004	Victoria	
2006	Southern	B/Malaysia/2506/2004	Victoria	
2005-2006	Northern	B/Jiangsu/10/2003	Yamagata	Vacc0506N B Jiangsu 10 2003
2005	Southern	B/Shanghai/361/2002	Yamagata	Vacc0504N 05S B Shanghai 361 2002
2004-2005	Northern	B/Shanghai/361/2002	Yamagata	
2004	Southern	B/Hong Kong/330/2001	Victoria	Vacc04S B Hong Kong 330 2001

The composition of the TIV is recommended bi-annually by the WHO. The influenza B component is often decided by HI inhibition assay of the prevalent influenza B virus currently circulating. The last column of this table lists the abbreviation used for each vaccine strain throughout this report.

### Sequence and phylogenetic analysis of gene segment 8

**Figure 3:**
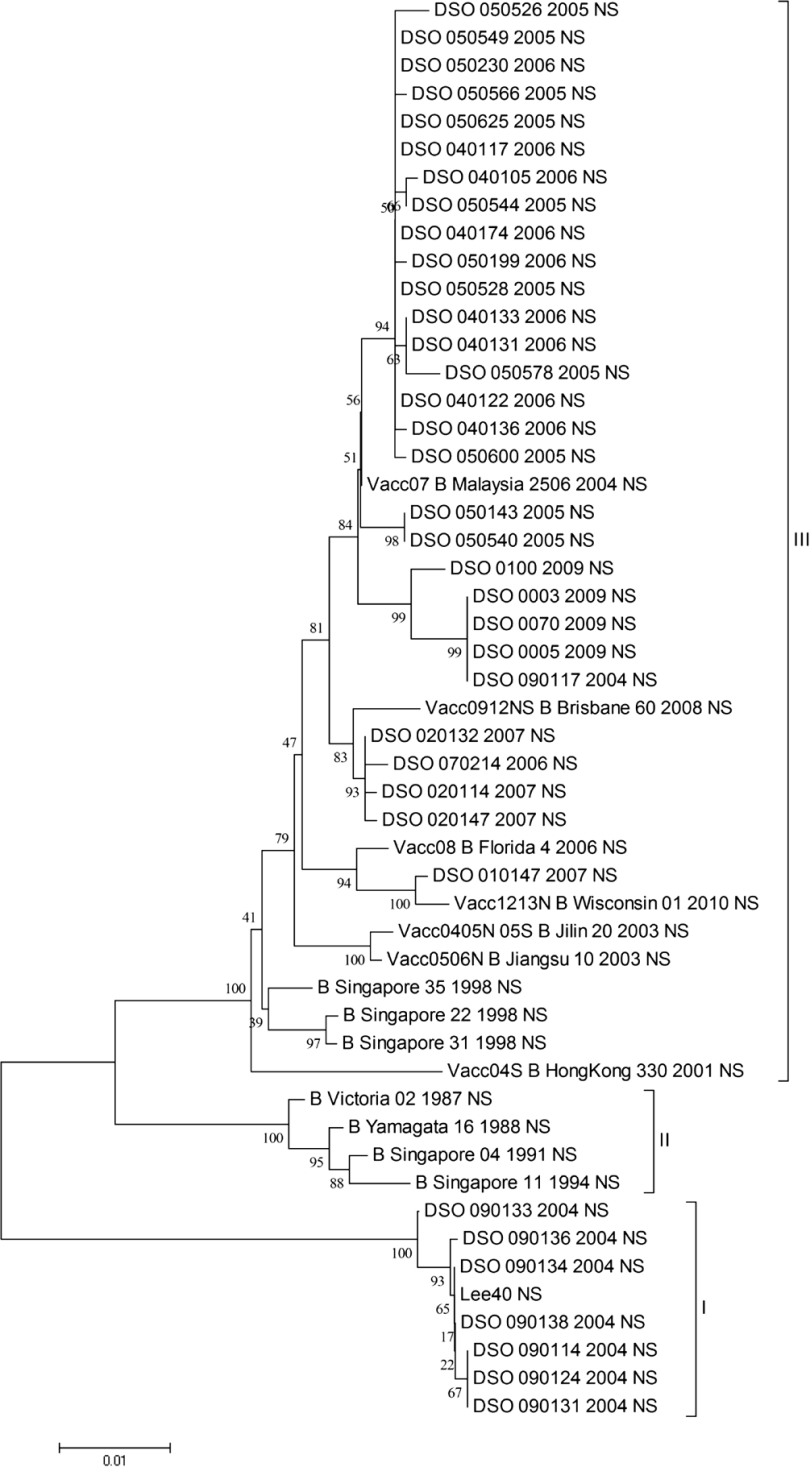
**Phylogenetic tree of the NS gene segment of Singaporean specimens isolated from 2004 to 2009.** The NS sequences of these specimens were aligned together with the NS gene segments of B/Lee/40, B/Yamagata/16/88, B/Victoria/2/87, the vaccine strains from 2004–2013 (Table 1) and the NA gene segments of Singaporean strains isolated in 1991, 1994 and 1998. Owing to the lack of NS gene sequences of recent strains from SEA, the NS alignment also included strains from the United States, Egypt and New Zealand which were isolated post 2000. The 3 different clusters of NS1 were labelled Clades I, II and III.

To facilitate future functional analysis, gene segment 8 was sequenced as described in the methods section. The 36 clinical specimens yielding sequence for gene segment 8 in this study displayed 89.8%-100% similarity. A phylogenetic tree of gene segment 8 was generated similarly to gene segments 4 and 6 (Figure 3). As reported previously, gene segment 8 of B/Victoria/2/87 and B/Yamagata/16/88 did not split into 2 separate clusters, unlike gene segments 4 and 6, in Figures 1 and 2 [25]. Instead, both strains fell under cluster II, suggesting similar ancestry (Figure 3) [25, 35–37]. The majority of the Singaporean specimens clustered in cluster III, together with most of the regional specimens and the vaccine strains. Interestingly, 7 of the specimens isolated in 2004 clustered together with B/Lee/40 in cluster I (Figure 3). This is the only instance where a gene segment clusters with B/Lee/40 (Figures 1, 2 and 3).

### HA protein sequence analysis

The main neutralising epitope of HA of the Victoria-like strains lies between residues 178–185 and this is known as the ‘tip’ (Table 2) [38]. The amino acid sequence of the specimens sequenced in this study as well as other Singaporean strains of Victoria lineage is identical to B/Victoria/87. Only two substitutions were observed in the vaccine strains: B/Hong Kong/330/2001 (E179D) and B/Brisbane/60/2008 (N180K). The ‘tip’ epitope is not as conserved amongst the Singapore strains of the Yamagata lineage (Table 2). For example, DSO_010147_2007, the only Yamagata-like specimen sequenced in this study has a N181Y substitution which was previously unseen in any sequence but in the vaccine strain B/Wisconsin/01/2010, suggesting that this substitution was newly introduced.

**Table 2:** **Amino acid substitution of the HA ‘Tip’ epitope**

	Yamagata ‘loop’								
	156								164
**Yamagata 88**	**G**	**S**	**C**	**P**	**N**	**V**	**T**	**S**	**R**
DSO 010147 2007	.	.	.	.	.	A	.	.	K
Vacc0405N 05S B Jilin 20 2003	.	.	.	.	.	A	.	.	K
Vacc0506N B Jiangsu 10 2003	R	.	.	.	.	A	.	.	K
Vacc0809NS B Florida 4 2006	.	.	.	.	.	A	.	.	K
Vacc1213N B Wisconsin 01 2010	.	.	.	.	.	A	.	.	K
Singapore 2010-2011	.	.	.	.	.	A	.	.	K
B Singapore 04 1991	.	.	.	.	.	.	.	N	.
B Singapore 11 1994	.	.	.	.	.	A	.	.	.
B Singapore 22 1998	.	.	.	.	.	A	.	.	K
B Singapore 31 1998	.	.	.	.	.	A	.	.	K
B Singapore 35 1998	.	.	.	.	.	A	.	.	.
B Singapore 222 1979	.	.	.	.	.	.	.	N	G
B Singapore 1964	.	.	.	.	.	.	.	N	G
**Victoria 87**	**G**	**S**	**C**	**P**	**N**	**V**	**T**	**N**	**G**
DSO Victoria	.	.	.	.	.	.	.	.	.
DSO 050629 2005	.	.	.	.	.	.	.	.	.
Vacc04S B Hong Kong 330 2001	.	.	.	.	.	.	.	.	.
Vacc0608NS B Malaysia 2506 2004	.	.	.	.	.	.	.	.	.
Vacc0912NS B Brisbane 60 2008	.	.	.	.	.	I	.	.	.
Singapore 2010-2011	.	.	.	.	.	.	.	.	.
Lee40	.	.	.	.	.	.	A	.	.

The ‘Tip’ epitope of the HA protein is the main neutralising epitope of viruses belonging to the Victoria lineage. Specimens listed under **B Yamagata 16 88** belong to the Yamagata lineage while specimens listed under **B Victoria 2 87** belong to the Victoria lineage. DSO Victoria: specimens sequenced in this study belonging to the Victoria lineage. Identical amino acids are marked by a dot (.) and deletions are denoted by a dash (−).

Table 3 lists the variation displayed at the main neutralising epitope of the HA from Yamagata-like strains known as the ‘loop’, between aa 156–164 [39]. DSO_010147_2007 has 2 substitutions in this epitope: V161A and R164K.

**Table 3:** **Amino acid substitution of the HA ‘Loop’ epitope**

	Victoria ‘tip’							
	178							185
**Yamagata 88**	˗	**D**	**N**	˗	**K**	**T**	**A**	**T**
DSO 010147 2007	˗	.	.	Y	.	N	.	.
Vacc0405N 05S B Jilin 20 2003	˗	.	.	.	.	N	.	.
Vacc0506N B Jiangsu 10 2003	˗	.	.	.	.	N	.	.
Vacc0809NS B Florida 4 2006	˗	.	.	.	.	N	.	.
Vacc1213N B Wisconsin 01 2010	˗	.	.	Y	.	N	.	.
Singapore 2010-2011	˗	.	.	Y	.	N	.	.
B Singapore 04 1991	˗	.	.	-	.	.	.	.
B Singapore 11 1994	˗	.	.	N	.	.	.	.
B Singapore 22 1998	˗	.	.	N	.	.	.	.
B Singapore 31 1998	˗	.	.	N	.	.	.	.
B Singapore 35 1998	˗	.	.	N	.	.	.	.
B Singapore 222 1979	˗	.	˗	N	.	.	.	.
B Singapore 1964	˗	N	K	N	.	.	.	R
**Victoria 87**	**N**	**D**	**N**	**N**	**K**	**T**	**A**	**T**
DSO Victoria	.	.	.	.	.	.	.	.
DSO 050629 2005	.	.	.	.	.	.	.	.
Vacc04S B Hong Kong 330 2001	.	E	.	.	.	.	.	.
Vacc0608NS B Malaysia 2506 2004	.	.	.	.	.	.	.	.
Vacc0912NS B Brisbane 60 2008	.	.	K	.	.	.	.	.
Singapore 2010-2011	.	.	K	.	.	.	.	.
Lee40	˗	.	.	.	.	.	.	I

The ‘Loop’ epitope of the HA protein is the main neutralising epitope of viruses belonging to the Yamagata lineage. Specimens listed under **B Yamagata 16 88** belong to the Yamagata lineage while specimens listed under **B Victoria 2 87** belong to the Victoria lineage. DSO Victoria: specimens sequenced in this study belonging to the Victoria lineage. Identical amino acids are marked by a dot (.) and deletions are denoted by a dash (−).

A glycosylation site at aa 211–213 (NET) was observed in most of the specimens isolated in Japan post 2002. Prior to 2002, aa 211–213 displayed a variety of sequences (NEA, KET, NEN and NET) [40]. Table 4 lists the sequence of the strains analysed in this study. All of the specimens sequenced had this glycosylation site except for DSO_050629_2005 (NEI). Interestingly, the HA gene segment of DSO_050629_2005 belonged to the Victoria lineage and this glycosylation site was initially found only in Japanese strains belonging to the same lineage. The same glycosylation site was observed even in Singaporean specimens isolated in 2010–2011. B/Singapore/222/1979 and B/Singapore/1964 did not have this glycosylation site (TET and NEI), while, 3 of the 5 Singaporean specimens isolated in the 90s contained the glycosylation site (NKT) sequences.

**Table 4:** **Amino acid substitution of a potential glycosylation site at position 211-213**

	HA glycosylation site		
	211		213
**Yamagata 88**	**D**	**K**	**T**
DSO 010147 2007	N	.	.
Vacc0405N 05S B Jilin 20 2003	N	.	.
Vacc0506N B Jiangsu 10 2003	N	.	.
Vacc0809NS B Florida 4 2006	.	.	.
Vacc1213N B Wisconsin 01 2010	N	.	.
Singapore 2010-2011	N	.	.
B Singapore 04 1991	N	.	I
B Singapore 11 1994	N	.	.
B Singapore 22 1998	N	.	N
B Singapore 31 1998	N	.	.
B Singapore 35 1998	N	.	.
B Singapore 222 1979	T	E	.
B Singapore 1964	N	E	I
**Victoria 87**	**N**	**E**	**A**
DSO Victoria	.	.	T
DSO 050629 2005	.	.	I
Vacc04S B Hong Kong 330 2001	S	.	T
Vacc0608NS B Malaysia 2506 2004	.	.	X
Vacc0912NS B Brisbane 60 2008	.	.	T
Singapore 2010-2011	.	.	T
Lee40	**D**	**K**	**T**

Specimens listed under **B Yamagata 16 88** belong to the Yamagata lineage while specimens listed under **B Victoria 2 87** belong to the Victoria lineage. DSO Victoria: specimens sequenced in this study belonging to the Victoria lineage. Identical amino acids are marked by a dot (.) and X refers to an unknown amino acid.

Three separate epitopes on the HA of influenza B have been identified which are targets of binding of antibodies CR8033, CR8071 and CR9114. These three antibodies were able to provide immunological protection in mice when administered after influenza B viral infection in mice [41]. The epitope recognised by CR8033 exists on the HA trimer, which overlaps the conserved receptor binding site (Table 5). Specimens of Yamagata lineage displayed variability at positions 151, 165, 177, 215 and 218, while only two specimens of the Victoria lineage showed variability at position 151. Uniquely, while N165 was conserved in all the Victoria strains, the Yamagata strains listed in Table 5 could harbour an Isoleucine, Serine, Aspartic Acid or a Lysine. Similarly, N218 was conserved in Victoria strains but variability was observed in the Yamagata strains (Serine, Lysine and Threonine) (Table 5). R177 was conserved in Singaporean Yamagata strains isolated between 1991 and 1998, while specimens before and after that period contained a Lysine. Notably, P176, which when substituted to a Glutamic acid resulted to poor susceptibility to CR8033, was conserved in all the specimens listed in Table 5.

**Table 5:** **Amino acid substitution of the CR8033 epitope**

	CR8033 (Head)												
	154	155	157	165	175	177	178	215	218	219	255	256	258
**Yamagata 88**	**T**	**S**	**S**	**N**	**V**	**R**	**-**	**Q**	**N**	**L**	**L**	**P**	**S**
DSO 010147 2007	**.**	**.**	**.**	**I**	**.**	**K**	**.**	**.**	**.**	**.**	**.**	**.**	**.**
Vacc0405N 05S B Jilin 20 2003	**.**	**.**	**.**	**S**	**.**	**K**	**.**	**P**	**.**	**.**	**.**	**.**	**.**
Vacc0506N B Jiangsu 10 2003	**.**	**.**	**.**	**S**	**.**	**K**	**.**	**.**	**.**	**.**	**.**	**.**	**.**
Vacc0809NS B Florida 4 2006	**.**	**.**	**.**	**S**	**.**	**K**	**.**	**.**	**.**	**.**	**.**	**.**	**.**
Vacc1213N B Wisconsin 01 2010	**.**	**.**	**.**	**I**	**.**	**K**	**.**	**.**	**S**	**.**	**.**	**.**	**.**
Singapore 2010–2011 (Yam)	**.**	**.**	**.**	**I**	**.**	**K**	**.**	**.**	**.**	**.**	**.**	**.**	**.**
B Singapore 04 1991	**.**	**.**	**.**	**D**	**.**	**.**	**.**	**.**	**.**	**.**	**.**	**.**	**.**
B Singapore 11 1994	**.**	**.**	**.**	**S**	**.**	**.**	**.**	**.**	**.**	**.**	**.**	**.**	**.**
B Singapore 22 1998	**.**	**.**	**.**	**S**	**.**	**.**	**.**	**.**	**.**	**.**	**.**	**.**	**.**
B Singapore 31 1998	**.**	**.**	**.**	**S**	**.**	**.**	**.**	**.**	**.**	**.**	**.**	**.**	**.**
B Singapore 35 1998	**.**	**.**	**.**	**S**	**.**	.	**.**	**.**	**.**	**.**	**.**	**.**	**.**
B Singapore 222 1979	**.**	**.**	**.**	**N**	**.**	**K**	**.**	**.**	**K**	**.**	**.**	**.**	**.**
B Singapore 1964	**.**	**.**	**.**	**K**	**.**	**K**	**.**	**.**	**T**	**.**	**.**	**Q**	**.**
**Victoria 87**	**T**	**S**	**S**	**N**	**V**	**K**	**N**	**Q**	**K**	**L**	**L**	**P**	**S**
DSO Victoria	**.**	**.**	**.**	**.**	**.**	**.**	**.**	**.**	**.**	**.**	**.**	**.**	**.**
Vacc04S B Hong Kong 330 2001	**.**	**.**	**.**	**.**	**.**	**.**	**.**	**.**	**.**	**.**	**.**	**.**	**.**
Vacc0608NS B Malaysia 2506 2004	**.**	**.**	**.**	**.**	**.**	**.**	**.**	**.**	**.**	**.**	**.**	**.**	**.**
Vacc0912NS B Brisbane 60 2008	**.**	**.**	**.**	**.**	**.**	**.**	**.**	**.**	**.**	**.**	**.**	**.**	**.**
Singapore 2010–2011 (Vic)	**.**	**.**	**.**	**.**	**.**	**.**	**.**	**.**	**.**	**.**	**.**	**.**	**.**
**Lee40**	**T**	**S**	**S**	**N**	**I**	**K**	**-**	Q	R	**L**	**L**	**K**	**S**

Specimens listed under **B Yamagata 16 88** belong to the Yamagata lineage while specimens listed under **B Victoria 2 87** belong to the Victoria lineage. DSO Victoria: specimens sequenced in this study belonging to the Victoria lineage. Identical amino acids are marked by a dot (.) and X refers to an unknown amino acid.

A K53E substitution within the CR8071 epitope has been shown to produce mutant viruses with reduced susceptibility to the antibody. This substitution was not observed in any of the specimens listed except that DSO_010151_2005 had an Arginine at this position. This substitution does not result in charge reversal as in K53E and may not result in any significant antigenic change (Table 6). It has also been noted that strains with a Histidine at position 40 rather than a Tyrosine are not effectively neutralized by CR8071. Antibody CR9114 binds to a conserved region within the stem region of the HA protein of both influenza A and B viruses [41]. Singaporean specimens isolated in 2010 and 2011 of the Yamagata lineage displayed K323R and DSO_050599_2005 of the Victoria lineage, displayed I380V (Table 7).

**Table 6:** **Amino acid substitution of the CR8071 epitope**

	CR8071 (Head)																	
	52	53	55	56	67	73	74	75	76	77	100	101	105	300	301	302	303	305
**Yamagata 88**	**T**	**K**	**H**	**F**	**K**	**L**	**N**	**C**	**C**	**T**	**H**	**E**	**V**	**G**	**S**	**L**	**P**	**I**
DSO 010147 2007	**.**	**.**	**Y**	**.**	**.**	**.**	**.**	**.**	**.**	**.**	**.**	**.**	**.**	**.**	**.**	**.**	**.**	**.**
Vacc0405N 05S B Jilin 20 2003	**.**	**.**	**Y**	**.**	**.**	**.**	**.**	**.**	**.**	**.**	**.**	**.**	**.**	**.**	**.**	**.**	**.**	**.**
Vacc0506N B Jiangsu 10 2003	**.**	**.**	**Y**	**.**	**.**	**.**	**.**	**.**	**.**	**.**	**.**	**.**	**.**	**.**	**.**	**.**	**.**	**.**
Vacc0809NS B Florida 4 2006	**.**	**.**	**Y**	**.**	**.**	**.**	**.**	**.**	**.**	**.**	**.**	**.**	**.**	**.**	**.**	**.**	**.**	**.**
Vacc1213N B Wisconsin 01 2010	**.**	**.**	**Y**	**.**	**.**	**.**	**.**	**.**	**.**	**.**	**.**	**.**	**.**	**.**	**.**	**.**	**.**	**.**
Singapore 2010-2011	**.**	**.**	**Y**	**.**	**.**	**.**	**.**	**.**	**.**	**.**	**.**	**.**	**.**	**.**	**.**	**.**	**.**	**.**
B Singapore 04 1991	**.**	**.**	**.**	**.**	**.**	**.**	**.**	**.**	**.**	**.**	**.**	**.**	**.**	**.**	**.**	**.**	**.**	**.**
B Singapore 11 1994	**.**	**.**	**.**	**.**	**.**	**.**	**.**	**.**	**.**	**.**	**.**	**.**	**.**	**.**	**.**	**.**	**.**	**.**
B Singapore 22 1998	**.**	**.**	**Y**	**.**	**.**	**.**	**.**	**.**	**.**	**.**	**.**	**.**	**.**	**.**	**.**	**.**	**.**	**.**
B Singapore 31 1998	**.**	**.**	**.**	**.**	**.**	**.**	**.**	**.**	**.**	**.**	**.**	**.**	**.**	**.**	**.**	**.**	**.**	**.**
B Singapore 35 1998	**.**	**.**	**.**	**.**	**.**	**.**	**.**	**.**	**.**	**.**	**.**	**.**	**.**	**.**	**.**	**.**	**.**	**.**
B Singapore 222 1979	**.**	**.**	**.**	**.**	**.**	**.**	**.**	**.**	**.**	**.**	**.**	**.**	**.**	**.**	**.**	**.**	**.**	**.**
B Singapore 1964	**.**	**.**	**.**	**.**	**.**	**.**	**.**	**.**	**.**	**.**	**.**	**.**	**.**	**.**	**.**	**.**	**.**	**.**
**Victoria 87**	**T**	**K**	**H**	**F**	**K**	**L**	**N**	**C**	**C**	**T**	**H**	**E**	**V**	**G**	**S**	**L**	**P**	**I**
DSO Victoria	**.**	**.**	**.**	**.**	**.**	**.**	**.**	**.**	**.**	**.**	**.**	**.**	**.**	**.**	**.**	**.**	**.**	**.**
DSO Victoria 2009	**I**	**.**	**.**	**.**	**.**		**.**	**.**	**.**	**.**	**.**	**.**	**.**	**.**	**.**	**.**	**.**	**.**
DSO 010151 2005	**.**	**R**	**.**	**.**	**.**	**.**	**.**	**.**	**.**	**.**	**.**	**.**	**.**	**.**	**.**	**.**	**.**	**.**
DSO 090114 2004	**.**	**.**	**.**	**.**	**.**	**F**	**.**	**.**	**.**	**.**	**.**	**.**	**.**	**.**	**.**	**.**	**.**	**.**
Vacc04S B Hong Kong 330 2001	**.**	**.**	**.**	**.**	**.**	**.**	**.**	**.**	**.**	**.**	**.**	**.**	**.**	**.**	**.**	**.**	**.**	**.**
Vacc0608NS B Malaysia 2506 2004	**.**	**.**	**.**	**.**	**.**	**.**	**.**	**.**	**.**	**.**	**.**	**.**	**.**	**.**	**.**	**.**	**.**	**.**
Vacc0912NS B Brisbane 60 2008	**.**	**.**	**.**	**.**	**.**	**.**	**.**	**.**	**.**	**.**	**.**	**.**	**.**	**.**	**.**	**.**	**.**	**.**
B Singapore 1H 2010	**.**	**.**	**.**	**.**	**.**	**P**	**.**	**.**	**.**	**.**	**.**	**.**	**.**	**.**	**.**	**.**	**.**	**.**
B Singapore 17H 2010	**.**	**.**	**.**	**.**	**.**	**P**	**.**	**.**	**.**	**.**	**.**	**.**	**.**	**.**	**.**	**.**	**.**	**.**
B Singapore 32C 2010	**.**	**.**	**.**	**.**	**.**	**P**	**.**	**.**	**.**	**.**	**.**	**.**	**.**	**.**	**.**	**.**	**.**	**.**
B Singapore 57H 2010	**.**	**.**	**.**	**.**	**.**	**P**	**.**	**.**	**.**	**.**	**.**	**.**	**.**	**.**	**.**	**.**	**.**	**.**
B Singapore 65H 2010	**.**	**.**	**.**	**.**	**.**	**P**	**.**	**.**	**.**	**.**	**.**	**.**	**.**	**.**	**.**	**.**	**.**	**.**
B Singapore 71H 2010	**.**	**.**	**.**	**.**	**.**	**P**	**.**	**.**	**.**	**.**	**.**	**.**	**.**	**.**	**.**	**.**	**.**	**.**
B Singapore 77C 2011	**.**	**.**	**.**	**.**	**.**	**.**	**.**	**.**	**.**	**.**	**.**	**.**	**.**	**.**	**.**	**.**	**.**	**.**
B Singapore 90C 2011	**.**	**.**	**.**	**.**	**.**	**P**	**.**	**.**	**.**	**.**	**.**	**.**	**.**	**.**	**.**	**.**	**.**	**.**
**Lee40**	**T**	**K**	**H**	**F**	**K**	**F**	**N**	**C**	**C**	**T**	**H**	**E**	**A**	**G**	**S**	**L**	**P**	**I**

Specimens listed under **B Yamagata 16 88** belong to the Yamagata lineage while specimens listed under **B Victoria 2 87** belong to the Victoria lineage. DSO Victoria: specimens sequenced in this study belonging to the Victoria lineage. Identical amino acids are marked by a dot (.) amino acid.

**Table 7:** **Amino acid substitution of the CR9117 epitope**

	CR9117 (Stalk)																				
	43	45	46	47	321	322	323	380	381	382	383	398	400	403	404	407	408	410	411	414	418
**Yamagata 88**	**G**	**I**	**P**	**L**	**S**	**K**	**P**	**I**	**A**	**G**	**W**	**A**	**L**	**T**	**Q**	**I**	**N**	**I**	**T**	**L**	**S**
DSO 010147 2007	**.**	**.**	**.**	**.**	**.**	**.**	**.**	**.**	**.**	**.**	**.**	**.**	**.**	**.**	**.**	**.**	**.**	**.**	**.**	**.**	**.**
Vacc0405N 05S B Jilin 20 2003	**.**	**.**	**.**	**.**	**.**	**.**	**.**	**.**	**.**	**.**	**.**	**.**	**.**	**.**	**.**	**.**	**.**	**.**	**.**	**.**	**.**
Vacc0506N B Jiangsu 10 2003	**.**	**.**	**.**	**.**	**.**	**.**	**.**	**.**	**.**	**.**	**.**	**.**	**.**	**.**	**.**	**.**	**.**	**.**	**.**	**.**	**.**
Vacc0809NS B Florida 4 2006	**.**	**.**	**.**	**.**	**.**	**.**	**.**	**.**	**.**	**.**	**.**	**.**	**.**	**.**	**.**	**.**	**.**	**.**	**.**	**.**	**.**
Vacc1213N B Wisconsin 01 2010	**.**	**.**	**.**	**.**	**.**	**.**	**.**	**.**	**.**	**.**	**.**	**.**	**.**	**.**	**.**	**.**	**.**	**.**	**.**	**.**	**.**
Singapore 2010-2011	**.**	**.**	**.**	**.**	**.**	**R**	**.**	**.**	**.**	**.**	**.**	**.**	**.**	**.**	**.**	**.**	**.**	**.**	**.**	**.**	**.**
B Singapore 04 1991	**.**	**.**	**.**	**.**	**.**	**.**	**.**	**.**	**.**	**.**	**.**	**.**	**.**	**.**	**.**	**.**	**.**	**.**	**.**	**.**	**.**
B Singapore 11 1994	**.**	**.**	**.**	**.**	**.**	**.**	**.**	**.**	**.**	**.**	**.**	**.**	**.**	**.**	**.**	**.**	**.**	**.**	**.**	**.**	**.**
B Singapore 22 1998	**.**	**.**	**.**	**.**	**.**	**.**	**.**	**.**	**.**	**.**	**.**	**.**	**.**	**.**	**.**	**.**	**.**	**.**	**.**	**.**	**.**
B Singapore 31 1998	**.**	**.**	**.**	**.**	**.**	**.**	**.**	**.**	**.**	**.**	**.**	**.**	**.**	**.**	**.**	**.**	**.**	**.**	**.**	**.**	**.**
B Singapore 35 1998	**.**	**.**	**.**	**.**	**.**	**.**	**.**	**.**	**.**	**.**	**.**	**.**	**.**	**.**	**.**	**.**	**.**	**.**	**.**	**.**	**.**
B Singapore 222 1979	**.**	**.**	**.**	**.**	**.**	**.**	**.**	**.**	**.**	**.**	**.**	**.**	**.**	**.**	**.**	**.**	**.**	**.**	**.**	**.**	**.**
B Singapore 1964	**.**	**.**	**.**	**.**	**.**	**.**	**.**	**.**	**.**	**.**	**.**	**.**	X	X	X	X	X	X	X	X	X
**Victoria 87**	**G**	**I**	**P**	**L**	**S**	**K**	**P**	**I**	**A**	**G**	**W**	**A**	**L**	**T**	**Q**	**I**	**N**	**I**	**T**	**L**	**S**
DSO Victoria	**.**	**.**	**.**	**.**	**.**	**.**	**.**	**.**	**.**	**.**	**.**	**.**	**.**	**.**	**.**	**.**	**.**	**.**	**.**	**.**	**.**
DSO 050629 2005	**.**	**.**	**.**	**.**	**.**	**.**	**.**	**.**	**.**	**.**	**.**	**.**	**.**	**.**	**.**	**.**	**.**	**.**	**.**	**.**	**.**
DSO 050599 2005	**.**	**.**	**.**	**.**	**.**	**.**	**.**	**V**	**.**	**.**	**.**	**.**	**.**	**.**	**.**	**.**	**.**	**.**	**.**	**.**	**.**
Vacc04S B Hong Kong 330 2001	**.**	**.**	**.**	**.**	**.**	**.**	**.**	**.**	**.**	**.**	**.**	**.**	**.**	**.**	**.**	**.**	**.**	**.**	**.**	**.**	**.**
Vacc0608NS B Malaysia 2506 2004	**.**	**.**	**.**	**.**	**.**	**.**	**.**	**.**	**.**	**.**	**.**	**.**	**.**	**.**	**.**	**.**	**.**	**.**	**.**	**.**	**.**
Vacc0912NS B Brisbane 60 2008	**.**	**.**	**.**	**.**	**.**	**.**	**.**	**.**	**.**	**.**	**.**	**.**	**.**	**.**	**.**	**.**	**.**	**.**	**.**	**.**	**.**
Singapore 2010-2011	**.**	**.**	**.**	**.**	**.**	**.**	**.**	**.**	**.**	**.**	**.**	**.**	**.**	**.**	**.**	**.**	**.**	**.**	**.**	**.**	**.**
**Lee40**	**G**	**I**	**P**	**L**	**S**	**K**	**P**	**I**	**A**	**G**	**W**	**A**	**L**	**T**	**Q**	**I**	**N**	**I**	**T**	**L**	**S**

Specimens listed under **B Yamagata 16 88** belong to the Yamagata lineage while specimens listed under **B Victoria 2 87** belong to the Victoria lineage. DSO Victoria: specimens sequenced in this study belonging to the Victoria lineage. Identical amino acids are marked by a dot (.) and X information unavailable.

### NA protein sequence analysis

An important functional site located on the NA protein is the calcium binding site which lies between aa 318–350 [42]. Amino acid substitutions in the calcium binding site are listed in Table 8. Most of the substitutions listed are conservative, (E320D, D329N, and R346S) suggesting a positive selection for this site. For example, E320D is seen in five other specimens, B/Myanmar/M107/2007, B/Myanmar/M254/2007, B/Taiwan/14/2007, B/Taiwan/2894/2006 and B/Hong Kong/259/2010. D329 is observed in B/Malaysia/27127/2004, B/Malaysia/1899839/2007, B/Malaysia/1919534/2008, B/Taiwan/2171/2004 and B/Taiwan/71523/2007. K343E is observed in three specimens (B/Singapore/68C/2010, B/Singapore/83C/2011 and B/Singapore/87H/2011). R345S substitution is seen in the rest of the specimens analysed in the NA phylogenetic tree except for B/Bangkok/141/1994 which harboured a Leucine at that same position. Each of the amino acids listed are hydrophilic and are substituted with another hydrophilic residue, except for R345L in B/Bangkok/141/1994.

**Table 8:** **Amino acid substitution of the NA calcium binding site**

	NA calcium binding site			
	320	329	343	345
**B Yamagata 16 1988**	**E**	**D**	**K**	**R**
Rest of DSO Specimens	**.**	**.**	**.**	S
DSO 050528 2005	D	**.**	**.**	S
DSO 070214 2006	D	**.**	**.**	S
DSO 020114 2007	D	**.**	**.**	S
DSO 020132 2007	D	**.**	**.**	S
DSO 020147 2007	D	**.**	**.**	S
DSO 010147 2007	D	N	**.**	S
B Singapore 1H 2010	D	**.**	**.**	S
B Singapore 32C 2010	D	**.**	**.**	S
B Singapore 17H 2010	D	**.**	**.**	S
B Singapore 57H 2010	D	**.**	**.**	S
B Singapore 65H 2010	D	**.**	**.**	S
B Singapore 68C 2010	**.**	N	E	S
B Singapore 77C 2011	D	**.**	**.**	S
B Singapore 71H 2011	D	**.**	**.**	S
B Singapore 90C 2011	N	**.**	**.**	S
B Singapore 83C 2011	**.**	N	E	S
B Singapore 87H 2011	**.**	N	E	S
B Singapore 04 1991	**.**	**.**	**.**	**.**
B Singapore 11 1994	**.**	**.**	**.**	S
B Singapore 22 1998	**.**	**.**	**.**	S
B Singapore 31 1998	**.**	**.**	**.**	S
B Singapore 35 1991	**.**	**.**	**.**	S
B Singapore 222 1979	**.**	**.**	**.**	L
Vacc04S B Hong Kong 330 2001	**.**	**.**	**.**	S
Vacc0405N 05S B Jilin 20 2003	**.**	**.**	**.**	S
Vacc0405N 05S B Shanghai 361 2002	**.**	**.**	**.**	S
Vacc0608NS B Malaysia 2506 2004	**.**	**.**	**.**	S
Vacc0506N B Jiangsu 10 2003	**.**	**.**	**.**	S
Vacc0809NS B Florida 4 2006	**.**	N	**.**	S
Vacc0912NS B Brisbane 60 2008	D	N	**.**	S
Vacc1213N B Wisconsin 01 2010	**.**	N	**.**	S
**B Lee 40**	**K**	**D**	**K**	**L**
**B Victoria 02 1987**	**E**	**D**	**K**	**R**

4 amino acid positions within the calcium binding site display variability throughout the NA protein alignment; 320, 329, 343 and 345. Specimens listed under **B Yamagata 16 88** belong to the Yamagata. Rest of DSO Specimens: rest of specimens sequenced in this study not individually listed in this table. Identical amino acids are marked by a dot (.).

### NB protein sequence analysis

Gene segment 6 encodes for the short, hydrophobic NB protein, of which no function has yet been assigned. All of the amino acid substitutions within aa 19–40, the postulated transmembrane domain, were conservative, keeping this stretch hydrophobic (Table 9) [43–46].

**Table 9:** **Amino acid substitution of the NB protein**

	Transmembrane domain								Ecto domain			
	21	22	24	29	30	31	34	35	43	48	67	92
**B Yamagata 16 1988**	**V**	**I**	**T**	**F**	**T**	**V**	**T**	**V**	**F**	**N**	**P**	**L**
Rest of DSO Specimens	I	**.**	**.**	**.**	I	**.**	**.**	I	P	Y	**.**	.
DSO 070214 2006	I	**.**	**I**	**.**	I	**I**	**.**	I	**L**	**.**	**.**	**.**
DSO 050091 2006	I	**.**	**I**	**.**	I	**I**	**.**	I	**L**	**.**	**.**	**.**
DSO 020147 2007	I	**.**	**I**	**.**	I	**I**	**.**	I	**L**	**.**	**.**	**.**
DSO 020132 2007	I	**.**	**I**	**.**	I	**I**	**.**	I	**L**	**.**	**.**	**.**
DSO 020114 2007	I	**.**	**I**	**.**	I	**I**	**.**	I	**L**	**.**	**.**	**.**
DSO 020113 2007	I	**.**	**I**	**.**	I	**I**	**.**	I	**L**	**.**	**.**	**.**
DSO 050528 2005	I	**.**	**.**	**.**	I	**.**	**.**	I	P	Y	**.**	**F**
DSO 0003 2009	I	**.**	**.**	**.**	I	**.**	**.**	I	**.**	Y	**S**	**I**
DSO 0005 2009	I	**.**	**.**	**.**	I	**.**	**.**	I	**.**	Y	**S**	**I**
DSO 0070 2009	I	**.**	**.**	**.**	I	**.**	**.**	I	**.**	Y	**S**	**I**
DSO 0100 2009	I	**.**	**.**	**.**	I	**.**	**.**	I	**.**	Y	**S**	**I**
B Singapore 04 1991	**.**	T	**.**	**L**	**I**	**.**	**.**	**.**	**L**	Y	**.**	**.**
B Singapore 11 1994	A	**.**	**.**	**.**	.	**.**	**.**	**.**	**.**	Y	**.**	**.**
B Singapore 22 1998	**.**	**.**	**.**	**.**	.	**.**	I	I	**.**	Y	**.**	**.**
B Singapore 31 1998	**.**	**.**	**.**	**.**	.	**.**	I	I	**.**	Y	**.**	**.**
B Singapore 35 1991	**.**	**.**	**.**	**.**	.	**.**	**.**	I	**.**	Y	**.**	**.**
B Singapore 222 1979	**.**	**.**	**.**	**.**	.	**.**	**.**	**.**	**.**	Y	**.**	**.**
Vacc04S B Hong Kong 330 2001	**.**	**.**	**.**	L	.	**.**	**.**	**.**	**.**	**.**	**.**	**F**
Vacc0405N 05S B Jilin 20 2003	**.**	**.**	**.**	**.**	.	**.**	**.**	I	**.**	**.**	**.**	**.**
Vacc0405N 05S B Shanghai 361 2002	**.**	**.**	**.**	**.**	.	**.**	**.**	I	**.**	**.**	**.**	**.**
Vacc0608NS B Malaysia 2506 2004	I	**.**	**.**	**.**	I	**.**	**.**	I	**P**	**Y**	**.**	**.**
Vacc0506N B Jiangsu 10 2003	**.**	**.**	**.**	**.**	.	**.**	**.**	I	**.**	**.**	**.**	**.**
Vacc0809NS B Florida 4 2006	**.**	**.**	**.**	**.**	I	**.**	**.**	I	**.**	**.**	**.**	**.**
Vacc0912NS B Brisbane 60 2008	**.**	**.**	**.**	**.**	I	I	**.**	I	**L**	**.**	**.**	**.**
Vacc1213N B Wisconsin 01 2010	**.**	**.**	**.**	**.**	I	**.**	**.**	I	**.**	**.**	**.**	**.**
**B Lee 40**	**I**	**I**	**T**	**L**	**T**	**V**	**I**	**V**	**F**	**N**	**P**	**L**
**B Victoria 02 1987**	**V**	**I**	**T**	**L**	**I**	**V**	**T**	**V**	**F**	**N**	**P**	**L**

Amino acid 21–35 correspond to the putative transmembrane domain of the NB protein while positions 43–92 are located on the ecto domain of the protein. Specimens listed under **B Yamagata 16 88** belong to the Yamagata. Rest of DSO Specimens: rest of specimens sequenced in this study not individually listed in this table. Identical amino acids are marked by a dot (.).

### NS1 and NEP protein sequence analysis

Gene segment 8 encodes 2 proteins: the NS1 and the NEP proteins. The NS1 and NEP proteins of the seven specimens clustering in cluster I show similar amino acid profiles to B/Lee/40. These amino acid substitutions are not seen in the rest of the clinical specimens clustering in cluster III (Figure 3 and Tables 10 and 11). The nuclear localisation sequence (NLS) of the NS1 protein lies between aa 46–56 [47]. Within the NLS only two amino acid substitutions are observed (H49N and R53K). These substitutions are only seen in specimens of cluster I (Table 10).

**Table 10:** **Amino acid substitution of the NS1-NLS AND ISG15 binding site**

		NS1 nuclear localisation sequence										ISG15 binding site			
		46									56	34	97	101	
**I**	**B Lee 40**	**D**	**R**	**L**	**H**	**R**	**L**	**N**	**R**	**K**	**L**	**E**	**F**	**I**	**E**
	DSO 090114 2004	.	.	.	.	.	.	.	.	.	.	.	.	.	.
	DSO 090124 2004	.	.	.	.	.	.	.	.	.	.	.	.	.	.
	DSO 090131 2004	.	.	.	.	.	.	.	.	.	.	.	.	.	.
	DSO 090133 2004	.	.	.	.	.	.	.	.	.	.	.	.	.	.
	DSO 090134 2004	.	.	.	.	.	.	.	.	.	.	.	.	.	.
	DSO 090136 2004	.	.	.	.	.	.	.	.	.	.	.	.	.	.
	DSO 090138 2004	.	.	.	.	.	.	.	.	.	.	.	.	.	.
**II**	**B Yamagata 16 1988**	.	.	.	N	.	.	.	K	.	.	.	L	V	.
	**B Victoria 02 1987**	.	.	.	N	.	.	.	K	.	.	.	L	.	.
	B Singapore 04 1991	.	.	.	N	.	.	.	K	.	.	.	L	V	.
	B Singapore 11 1994	.	.	.	N	.	.	.	K	.	.	.	L	V	
**III**	DSO 090117 2004	.	.	.	N	.	.	.	K	.	.	.	L	.	.
	DSO 050143 2005	.	.	.	N	.	.	.	K	.	.	.	L	.	G
	DSO 050540 2005	.	.	.	N	.	.	.	K	.	.	.	L	.	G
	Rest of DSO Specimens	.	.	.	N	.	.	.	K	.	.	.	L	.	.
	B Singapore 22 1998	.	.	.	N	.	.	.	K	.	.	.	L	.	.
	B Singapore 31 1998	.	.	.	N	.	.	.	K	.	.	.	L	.	.
	B Singapore 35 1998	.	.	.	N	.	.	.	K	.	.	.	L	.	.
	Vacc04S B Hong Kong 330 2001	.	.	.	N	.	.	.	K	.	.	.	L	.	.
	Vacc0405N 05S B Jilin 20 2003	.	.	.	N	.	.	.	K	.	.	.	L	.	.
	Vacc0405N 05S B Shanghai 361 2002	.	.	.	N	.	.	.	K	.	.	.	L	.	.
	Vacc0608NS B Malaysia 2506 2004	.	.	.	N	.	.	.	K	.	.	.	L	.	.
	Vacc0506N B Jiangsu 10 2003	.	.	.	N	.	.	.	K	.	.	.	L	.	.
	Vacc0809NS B Florida 4 2006	.	.	.	N	.	.	.	K	.	.	.	L	.	.
	Vacc0912NS B Brisbane 60 2008	.	.	.	N	.	.	.	K	.	.	.	L	.	.
	Vacc1213N B Wisconsin 01 2010	.	.	.	N	.	.	.	K	.	.	.	L	.	.

Amino acids 46–56 correspond to the NLS of the NS1 protein. I: Specimens clustering in cluster I of the NS phylogenetic tree. II: Specimens clustering in cluster II of the NS phylogenetic tree. III: Specimens clustering in cluster III of the NS phylogenetic tree. Rest of DSO Specimens: rest of specimens sequenced in this study not individually listed in this table. Identical amino acids are marked by a dot (.).

**Table 11:** **Amino acid substitution of the NEP**

		NEP			
		35	66	80	98
**I**	**B Lee 40**	**S**	**A**	**S**	**I**
	DSO 090114 2004	.	.	.	.
	DSO 090124 2004	.	.	.	.
	DSO 090131 2004	.	.	.	.
	DSO 090133 2004	.	.	.	.
	DSO 090134 2004	.	.	.	.
	DSO 090136 2004	.	.	.	.
	DSO 090138 2004	.	.	.	.
**II**	**B Yamagata 16 1988**	.	V	N	.
	**B Victoria 02 1987**	.	V	N	.
	B Singapore 04 1991	.	V	N	.
	B Singapore 11 1994	.	V	N	.
**III**	DSO 090117 2004	N	V	N	V
	DSO 0003 2009	N	V	N	V
	DSO 0005 2009	N	V	N	V
	DSO 0070 2009	N	V	N	V
	Rest of DSO Seq	.	V	N	V
	B Singapore 22 1998	.	V	N	V
	B Singapore 31 1998	.	V	N	V
	B Singapore 35 1998	.	V	N	V
	B Singapore 04 1991	.	V	N	V
	Vacc04S B Hong Kong 330 2001	.	V	N	V
	Vacc0405N 05S B Jilin 20 2003	.	V	N	V
	Vacc0405N 05S B Shanghai 361 2002	.	V	N	V
	Vacc0608NS B Malaysia 2506 2004	.	V	N	V
	Vacc0506N B Jiangsu 10 2003	.	V	N	V
	Vacc0809NS B Florida 4 2006	.	V	N	V
	Vacc0912NS B Brisbane 60 2008	.	V	N	V
	Vacc1213N B Wisconsin 01 2010	.	V	N	V

3 amino acids of the NS1 protein (34, 97 and 101) within the ISG15 binding site display variability. I: Specimens clustering in cluster I of the NS phylogenetic tree. II: Specimens clustering in cluster II of the NS phylogenetic tree. III: Specimens clustering in cluster III of the NS phylogenetic tree. Rest of DSO Specimens: rest of specimens sequenced in this study not individually listed in this table. Identical amino acids are marked by a dot (.).

Influenza B NS1 has been shown to bind to and inhibit human ISG15 [48]. 19 amino acids in the first 101 residues of influenza B NS1 have been identified as directly interacting with ISG15 [49]. Out of these 19 amino acids, the specimens sequenced in this study displayed variation at positions 34, 97 and 101 (Table 10). Specimens of cluster I showed sequence identity to B/Lee/40 at these positions. F34L is seen in all the specimens in clades II and III, while I97V is only seen in the 3 specimens listed in Table 10 and in B/Bangkok/141/1994. E101G is only seen in DSO_050143_2005 and DSO_050540_2005.

### Amino acid substitution of the NEP

Amino acids of positions 35, 66, 80 and 98 of the NEP display sequence variability as listed. I: Specimens clustering in cluster I of the NS phylogenetic tree. II: Specimens clustering in cluster II of the NS phylogenetic tree. III: Specimens clustering in cluster III of the NS phylogenetic tree. Rest of DSO Specimens: rest of specimens sequenced in this study not individually listed in this table. Identical amino acids are marked by a dot (.).

Within the NEP protein, the S35N substitution is only seen in the specimens listed in Table 11 as well as B/Malaysia/33772/2005. While A66 was specific to the cluster I specimens, S80 was also observed in B/Hawaii/11/2004, B/Hawaii/13/2004 and B/Hawaii/33/2004. I98 was also present in B/Bangkok/141/1994 and B/Seoul/1163/2004 (Table 11).

## Discussion

There have been very few studies on influenza B virus epidemiology in Singapore. Among the earliest Singaporean strain sequenced, B/Singapore/1964, had gene segment 4 from the Yamagata lineage. No sequence information of gene segment 4 was available for this strain. Another early strain, B/Singapore/222/1979, had gene segment 4 belonging to the Yamagata lineage and gene segment 7 from the Victoria lineage. The Singaporean specimens isolated in the 1990s had both gene segments belonging to the Yamagata lineage (Figures 1 and 2). Reports of influenza B viruses isolated between 1990 and 1999, have suggested that the dominant lineage of that era (by HI-tests and sequencing of the HA gene alone) were from the Yamagata lineage, with few outbreaks of the Victoria lineage detected in Europe [4, 50–53]. The Victoria lineage, however, was suggested to be the emergent stain in 1987 in South China and Japan [5, 26].

Post 2004, the majority of the Singaporean specimens had gene segment 4 from the Victoria lineage and gene segment 6 from the Yamagata lineage; however a substantial minority had both gene segments from the Yamagata lineage, such as DSO_010147_2007. In a retrospective study, it was shown that prior to 2002, Yamagata-like viruses were dominant in South East Asia (SEA) [54]. During the year 2002, most of the viruses circulating in the same region were Victoria-like, while post-2002 both lineages have been circulating almost too equal frequencies. This suggests that viruses of the Victoria lineage could have emerged from South China and Japan and managed to spread to Singapore by 2002, as supported by previous studies [7, 8].

Vaccine studies in children as well as in laboratory animals had previously showed that the vaccination with the Yamagata lineage of influenza B does not provide immunity against the viruses of the Victoria lineage [55, 56]. The data from these studies also suggested that post 2000, influenza B viruses of both lineages were co-circulating simultaneously. This further suggests that including one influenza B strain in the bi-annual trivalent vaccine is clearly insufficient to protect the entire population against influenza B infection. For example, B/Malaysia/2506/2004, the vaccine strain suggested for 2006–2008, had gene segments 4 and 6 from the Victoria and Yamagata lineages respectively. Viruses of this re-assortment order represent the majority of the Singaporean specimens sequenced in this study. However, vaccinated individuals infected with viruses similar to DSO_010147_2007 would probably not be protected by the B/Malaysia/2506/2004 vaccine. The same case can be argued for the vaccine strain B/Brisbane/60/2008, which is similar in reassortment to B/Malaysia/2506/2004; but 3 out of the 11 Singaporean specimens isolated in 2010–2011 did not match this re-assortment pattern (Figures 1 and 2) further suggesting that the protection from the vaccine strain was not adequate. Similar observation was previously made where 95% of the influenza viruses circulating in 2007–2008 were of the Yamagata lineage while the vaccine strain was of the Victoria lineage [57]. In another report, it was shown that between the years 1999–2000, the circulating strains only match the vaccine strains in 5 out of the 10 years period [58]. The data from this study agrees with the latest injunction to include two strains of influenza B viruses in the biannual vaccine so as ensure that the public is adequately immunised by both lineages [58–60].

Gene segment 8 of B/Victoria/2/87 and B/Yamagata/16/88 clustered together in cluster II, suggesting similar ancestry (Figure 3). It is also possible that the split in the lineages occurred before the emergence of these two strains. Evidence for this lies in the clustering pattern of B/Singapore/222/1979 and B/Singapore/1964 in Figures 1 and 2. Both of these specimens were isolated prior to the emergence of the ancestor strains, yet still cluster within the Yamagata or Victoria lineages.

The V161A substitution in the HA protein has not been reported but strains carrying K164 have been shown to have the same reactivity with strains displaying R164. Strains displaying R156 and G164 have been documented with the inability to react with the antibody [39]. Interestingly vaccine strain B/Jiangsu/10/2003 has R156 while B/Singapore/222/1979 and B/Singapore/1964 have G164. Further functional studies will be required to determine if these two substitutions result in a change in antibody binding.

The data from Table 7, pertaining to the glycosylation site at aa 211–213, suggests that position 211 of the HA protein is probably exposed, and located on the exterior of the globular protein. The folding of the protein at this position is also independent of its lineage. All the vaccine strains except for B/Florida/4/2006 and B/Hong Kong/330/2001 did not contain this potential glycosylation site. The amino acid at position 213 for B/Malaysia/2506/2004 is unknown, as the codon at that position has the nucleotide sequence ayc (where y = c or t) therefore is it not possible to determine if this protein has the glycosylation site (Table 7). Further analysis of the specimens used to construct the HA phylogenetic tree revealed that all of the regional specimens contained this potential glycosylation site, except for B/Taiwan/72068/2004 (KKT), B/Taiwan/91061/2005 (SET), B/Taiwan/2894/2006 (DET), B/Taiwan/71523/2007 (SKT), B/Malaysia/1749642/2007 (SET) and B/Hong Kong/310/2004 (KET). It is possible that this glycosylation site originated in Japan prior to its spread worldwide; however since this glycosylation site was also seen in Singapore as early as 1994, it suggests that the origin of this glycosylation site occurred independent to the previous one. Studies in influenza A viruses have led to the suggestion that an introduction of a glycosylation site may mask an antibody-binding epitope, leading to an antigenic change [61]. This further suggests that a similar masking may take place at this glycosylation position for the influenza B virus HA protein.

The data from Table 6 suggests that CR8071 would be effective in neutralizing current specimens of the Yamagata lineage rather than the Victoria lineage. Position 73 also displayed sequence variability only amongst the specimens of the Victoria lineage, where specimens displayed a Leucine, Phenylalanine or Proline, further investigations have to be carried out to determine if any change in antibody binding would result (Table 6).

Both substitutions listed in Table 7 have not been previously reported but their conservative nature, suggests there might not be any change in antibody binding. This conserved stem region shown in several reports and ours suggests that vaccines eliciting antibodies against such epitopes may provide long lasting protection against both influenza A and B viruses [41, 62] (Table 7).

This K343E substitution observed in the NA protein is interesting as it represents a reversal in the charge in a domain which requires conservation of charge for calcium ion binding, as evidenced by their difference in pKa values: Glutamic Acid-4.07 and Lysine-10.53. This difference in pKa could possibly affect calcium binding. The selection for the calcium binding site of the NA protein to remain hydrophilic strongly suggests conservation of phenotype (Table 8). However, functional and structural assays are needed in order to determine if these substitutions do have an actual effect on calcium binding.

None of the previously cited amino acid changes associated to neuraminidase inhibitor (NAI) resistance were found in this study in either the HA or NA proteins [33, 63–72].

While the function of the NB protein is still unknown, aa 19–40 have been shown to constitute its transmembrane domain [43–46]. The data from this study strengthens the view that the function of NB is membrane-associated since there seems to be a selective pressure in maintaining aa 19–40 hydrophobic (Table 9).

P67S and L92I in the NB were only seen in Singaporean strain isolated in 2009 (Table 9). Neither one of these substitutions occur independently of each other, suggesting that both these amino acids might act in concert in the function of NB.

While R53K in the NS1 protein is conservative, H49N results in the substitution of a basic amino acid to a neutrally charged amino acid (Table 10). This might result in a change in the ability of NS1 to localise to the nucleus. Further functional analysis has to be carried out to determine if the amino acid substitutions listed in Tables 10 and 11 result in a change of NS1 binding to ISG15 or NEP’s ability to transport vRNPs out of the nucleus (Tables 10 and 11).

## Conclusions

Phylogenetic analyses of clinical specimens reveal that majority of influenza B strains detected between the years of 2004–2009 were reassortants with gene segments 4 and 6 belonging to the Victoria and Yamagata lineages respectively. This data corroborates with the Singaporean specimens isolated in 2010–2011. Several vaccine mismatches were observed in years 2007, 2010 and 2011, strongly proving the need for a quadrivalent vaccine.

The NS1 gene of specimens detected in 2004 show a strong similarity to B/Lee/40 NS1, unseen in other specimens. This phenomenon could suggest that B/Yamagata/88 and B/Victoria/87 are not representative of the split in influenza B evolution and that a third cluster similar to B/Lee/40 is still in circulation, albeit being a minority.

No amino acid substitution relating to drug resistance has been identified in the specimens sequenced in this study. Other amino acid substitutions highlighted in this study require further functional analysis to determine their ability to affect the protein’s phenotype.

We believe, to the best of our knowledge, that this is the first study of influenza B epidemiology in South East Asia to focus on sequence analysis of gene segments 4, 6 and 8. Gene segments 4 and 6 were chosen as they encode the HA and NA proteins which are the most antigenic proteins of the virus, while gene segment 8 encodes the NS1 protein which is the main protein involved in pathogenesis. The clinical specimens analysed were sequenced directly from VTM and were not passaged through eggs or tissue culture to avoid tissue culture/egg adaptations. This avoids tissue culture/egg adaptations, giving us an accurate representation of the circulating sequences of influenza B viruses.

## Methods

### Virus

The influenza B virus, strain B/Lee/40 (VR101) was purchased from the American Type Culture Collection (ATCC). The virus was stored in 1 ml aliquots in −80°C. 12 day old embryonated chicken eggs were infected with B/Lee/40 for 48 hrs at 37°C, 5% CO_2_. Allantoic fluid was harvested and clarified at 2000 rpm for 15 min. 1 ml aliquots of the clarified allantoic fluid were stored in −80°C.

### Clinical specimens

Specimens were collected from the Singapore military men who reported sick to the medical centres of various camps between 2004 and 2009. Only individuals presenting a fever of greater than 38°C (oral temperature) with cough and/or sore throat were recruited for the study. Consenting patients displaying the abovementioned symptoms provided throat swabs or nasal swabs, which were then re-suspended in viral transport medium (VTM), as previously described [30]. These specimens are named according to the following format; DSO_(numerical code)_(year of collection). This study, reference 160D-7/404-3, was approved by the Joint Medical Committee, Research, of the Singapore Armed Forces [30].

### Extraction of influenza B virus vRNA

Viral RNA (vRNA) was extracted using the RNeasy minikit (Qiagen, Inc., Valencia, CA, USA) according to manufacturer’s instructions. The extracted vRNA were stored in −80°C until real-time PCR assays were performed as described in Seah et al., [30]. Only PCR-positive specimens were used for sequencing of the HA and NA genes.

### Primers

Table 12 lists all the primers used in this study. Primers were designed in-house by the alignment of HA, NA and NS gene segments of influenza B isolated in Singapore previously as available from NCBI database. Primers were designed using BioEdit Software (See Table 13).

**Table 12:** **Primers used in this study**

Primer name	5′ Sequence 3′	Amplicon size	Gene
	**Diagnostic Primers**		
FluB 970 F^♦^	AAATACGGTGGATTAAACAAAAGCAA	161 bp	HA
FluB 1131R^♦^	AGCTCCGAAGAAACCCCTTTCC		
FluB 1004 Probe^♦^	CACCCATATTGGGCAATTTCCTATGGC		
	**Sequencing Primers**		
UniB^*^	AGC AGA AGC	-	All
HA 30 F^§^	CTA CTC ATG GTA GTA ACA TCC	709 bp	HA
HA 749R^§^	YGG GAA GCC ACC AAT CTG AGA AAC		
HA 471 F^§^	ACC TCA GGA TCT TGC CCT AAC G	689 bp	
HA1169R^§^	TGT GTA TCC GTG CCA ACC TGC AAT		
HA 999 F^§^	AAA GCC ATA GGA AAT TGC CCA	841 bp	
HA 1840R^§^	TCA ATA ACG TTT CTT TGT AAT		
HA F^⊥^	TAT TCG TCT CAG GGA GCA GAA GCA GAG CAT TTT CTA ATA TC	1840 bp	
HA R^⊥^	ATA TCG CTC CGT ATT AGT AGT AAC AAG AGC ATT TTT C		
NA 21F^Δ^	GCT ACC TTC AAC TAT ACA AAC G	547 bp	NA
NA 568R^§^	TAC CAT CAT GGC ATG CGG A		
NA 361 F^§^	GCT CCC TTG ATA ATA AGG GAA CC	477 bp	
NA 838R^§^	ATG TTC TAC TCT TCC TGT TGG		
NA 716 F^§^	GGG GGA RAT TGT TAT CTT ATG	789 bp	
NA 1505R^§^	TTT CAG AAA CAA TTA AKT TCA GTA AGG		
NA F^⊥^	TAT TCG TCT CAG GGA GCA GAA GCA GAG CA	1505 bp	
NA R^⊥^	ATA TCG CTC CGT ATT AGT AGT AAC AAG AGC ATT TT		
NS 376R^§^	CTG GTG TTG AAG GGT AAT	-	NS
NS 700R^§^	AT CTT CTT CAT CCT CCA CTG TAA	-	
NS F^⊥^	TAT TCG TCT CAG GGA GCA GAA GCA GAG GAT TTG TTT AGT C	1053 bp	
NS R^⊥^	ATA TCG TCT CGT ATT AGT AGT AAC AAG AGG ATT TTT AT		

^*^Taken from Zou et al., [73].

^⊥^Taken from Hoffmann et al., 2000 [74].

^§^In-house designed primers.

^Δ^Taken from Tsai, 2006 [89].

^♦^Taken from Krafft et al., 2005 [90].

**Table 13:** **Touchdown PCR thermocycling conditions**

**Initial denaturation**		94°C	2 min	
**Touchdown**	11 cycles	94°C	30 sec	
		**55**°C	**30 sec**	**−1°C** **/cycle**
		68°C	2 min	
Denaturation	40 cycles	94°C	30 sec	
Annealing		4x8°C	30 sec	
Extension		68°C	2 min	
Final extension		68°C	10 min	
Holding		12°C	forever	

All the PCR conditions in this study are touchdown PCR, with adjustments to the annealing temperatures, and extension times.

### cDNA synthesis

Complementary DNA synthesis was carried out for the specimens using either the Transcriptor First-Strand cDNA synthesis System for RT-PCR (Roche) with the reverse transcription step carried out at 65°C for 30 minutes or the SuperScript II First-Strand cDNA Synthesis Kit (Invitrogen) with UniB primer (Table 12) [73] and with the reverse transcription step carried out at 42°C for 1 hour. Other than the modifications mentioned, all other steps were carried out according to manufacturer’s instructions.

### Hemagglutinin (HA) gene

The cDNA synthesized from above was used to PCR-amplify the full HA gene segment using primers designated HA F and HA R [74] (Table 12) with the Platinum Taq Polymerase High Fidelity kit (Invitrogen) using touchdown PCR thermocycling conditions: initial denaturation at 95C for 2 minutes, followed by 11 cycles of, 94C-30 sec, 55C-30 sec (−1C/cycle) and 68°C for 2 min and 40 cycles of 94°C-30 sec, 48°C-sec, 68°C-2 min, a final extension at 68°C for 10 minutes and holding temperature at 4°C. A second round of PCR was performed to obtain 3 overlapping fragments of the HA gene segment using the primers; HA30F/HA749R, HA471F/HA1169R and HA999/HA1840R (Table 12). The thermocycling conditions for the amplification of all three of these fragments are similar to that described above but with an extension step of 1 min.

### Neuraminidase (NA) gene

The full NA gene was PCR-amplified from cDNAs as described for the full HA gene; except that the primers used were NA F and NA R [74] (Table 1) using the same enzyme and thermocycling conditions described. Again, a second round of PCR was performed to obtain 3 overlapping fragments of the NA gene segment using the primers; NA21F/NA568R, NA361F/NA838R and NA716F/NA1505R (Table 12). The thermocycling conditions for each primer sets required annealing temperatures of 45°C, 58°C and 50°C respectively and all with an elongation time of 1 min.

### Non Structural (NS) gene

The full NS gene segment was amplified from the synthesized cDNA using primers NS F and NS R (Table 12) [74]. The thermocycling conditions required an annealing temperature of 62°C and an elongation temperature of 72°C for 10 minutes. The NS gene segment was sequenced directly using primers NSF, NSR as well as NS700R and NS376R (Table 12).

### Sequencing and bioinformatics

Eluted PCR products were sent to 1^st^ BASE Singapore for sequencing. Each PCR fragment was gel-extracted and purified before subjecting to sequencing using the ABI big dye with the respective PCR primers. The DNA sequences of each fragment were assembled using SeqMan program (DNASTAR). For each of the HA, NA and NS1 genes, the nucleotide sequences were aligned with Clustal W from MegAlign software (DNASTAR) and the phylogenetic trees were next generated using the Neighbour-Joining algorithm. The number of bootstrap replications was set to 1000 and bootstrap values were labelled on the tree branches [75]. The same software was used to locate the positions of the correct open reading frames (ORFs) and the amino acid sequences were next translated. Sequence analysis was performed with closely related sequences obtained with the NCBI blast search, and the vaccine strains for years 2004–2013 [9, 76–88] (Tables 7 and 8).

### Availability of supporting data

The nucleotide and translated amino acid sequences supporting the phylogenetic trees in this study have been included in the GenBank repository [http://www.ncbi.nlm.nih.gov/genbank] and the following accession numbers have been assigned GU943154-GU943235, KC844161-KC844196. The phylogenetic trees generated for this study have also been uploaded to the TreeBase repository with a submission ID of 16558 [http://purl.org/phylo/treebase/phylows/study/TB2:S16558].

## Authors’ information

TBH is head of the Diagnostics and Detection Laboratory (DDL) of DSO National Laboratories (Singapore). RJS is Head of Division of the Division of Molecular Genetics & Cell Biology of the School of Biological Sciences, Nanyang Technological University. MRJ is a PhD student at the School of Biological Sciences, Nanyang Technological University.

## Electronic supplementary material

Additional file 1: Table S1: Total of 46 clinical specimens included in this study and their genes which have been sequenced. * Specimens which gave sequence information for all three genes. (Y): Yamagata Lineage, (V): Victoria Lineage, (I): Clade I, (III): Clade III. (DOC 60 KB)
