# Supplementary material for: Genetic characterisation of influenza B viruses detected in Singapore, 2004 to 2009
Source: BMC Res Notes. 2014 Dec 1;7:863. doi: 10.1186/1756-0500-7-863 (PMC4265450; doi:10.1186/1756-0500-7-863)
Supplement: Supplementary file 1 — Additional file 1: Table S1: Total of 46 clinical specimens included in this study and their genes which have been sequenced. * Specimens which gave sequence information for all three genes. (Y): Yamagata Lineage, (V): Victoria Lineage, (I): Clade I, (III): Clade III. (DOC 60 KB) [file 13104_2014_3377_MOESM1_ESM.doc]

**Additional file 1: Table S1.** Total number of genes sequenced in this study.

| **Specimen Name** | Gene Segments Sequenced | | |
| --- | --- | --- | --- |
| DSO_090114_2004***** | HA (V) | NA (Y) | NS (I) |
| DSO_090117_2004 |  |  | NS (III) |
| DSO_090124_2004***** | HA (V) | NA (Y) | NS (I) |
| DSO_090131_2004***** | HA (V) | NA (Y) | NS (I) |
| DSO_090133_2004***** | HA (V) | NA (Y) | NS (I) |
| DSO_090134_2004***** | HA (V) | NA (Y) | NS (I) |
| DSO_090136_2004***** | HA (V) | NA (Y) | NS (I) |
| DSO_090138_2004 |  |  | NS (I) |
| DSO_010143_2005***** | HA (V) | NA (Y) | NS (III) |
| DSO_010151_2005 | HA (V) | NA (Y) |  |
| DSO_050526_2005***** | HA (V) | NA (Y) | NS (III) |
| DSO_050528_2005***** | HA (V) | NA (Y) | NS (III) |
| DSO_050540_2005***** | HA (V) | NA (Y) | NS (III) |
| DSO_050541_2005 | HA (V) | NA (Y) |  |
| DSO_050544_2005***** | HA (V) | NA (Y) | NS (III) |
| DSO_050549_2005***** | HA (V) | NA (Y) | NS (III) |
| DSO_050566_2005***** | HA (V) | NA (Y) | NS (III) |
| DSO_050578_2005***** | HA (V) | NA (Y) | NS (III) |
| DSO_050599_2005 | HA (V) | NA (Y) |  |
| DSO_050600_2005***** | HA (V) | NA (Y) | NS (III) |
| DSO_050619_2005 |  | NA (Y) |  |
| DSO_050622_2005 | HA (V) | NA (Y) |  |
| DSO_050625_2005***** | HA (V) | NA (Y) | NS (III) |
| DSO_050629_2005 | HA (V) | NA (Y) |  |
| DSO_050633_2005 | HA (V) | NA (Y) |  |
| DSO_040105_2006***** | HA (V) | NA (Y) | NS (III) |
| DSO_040117_2006***** | HA (V) | NA (Y) | NS (III) |
| DSO_040122_2006***** | HA (V) | NA (Y) | NS (III) |
| DSO_040131_2006***** | HA (V) | NA (Y) | NS (III) |
| DSO_040133_2006***** | HA (V) | NA (Y) | NS (III) |
| DSO_040136_2006 |  | NA (Y) | NS (III) |
| DSO_040174_2006***** | HA (V) | NA (Y) | NS (III) |
| DSO_040203_2006 | HA (V) | NA (Y) |  |
| DSO_050091_2006 |  | NA (Y) |  |
| DSO_050199_2006***** | HA (V) | NA (Y) | NS (III) |
| DSO_050230_2006***** | HA (V) | NA (Y) | NS (III) |
| DSO_070214_2006***** | HA (V) | NA (Y) | NS (III) |
| DSO_020113_2007 | HA (V) | NA (Y) |  |
| DSO_020114_2007***** | HA (V) | NA (Y) | NS (III) |
| DSO_020132_2007***** | HA (V) | NA (Y) | NS (III) |
| DSO_020147_2007***** | HA (V) | NA (Y) | NS (III) |
| DSO_010147_2007***** | HA (Y) | NA (Y) | NS (III) |
| DSO_0003_2009***** | HA (V) | NA (Y) | NS (III) |
| DSO_0005_2009***** | HA (V) | NA (Y) | NS (III) |
| DSO_0070_2009***** | HA (V) | NA (Y) | NS (III) |
| DSO_0100_2009***** | HA (V) | NA (Y) | NS (III) |
